# Smart Phone Chat Apps for Teaching and Assessment in Orthopaedic Residency Training

**DOI:** 10.5704/MOJ.2303.007

**Published:** 2023-03

**Authors:** WT Lavadia, EA Sana, MS Salvacion

**Affiliations:** 1 Section of Adult Orthopaedics, Philippine Orthopedic Center, Quezon City, Philippines; 2 National Teacher Training Center for the Health Professions, University of the Philippines Manila, Manila, Philippines

**Keywords:** e-learning, smart phone chat applications, residency training in orthopaedics, outcome-based education

## Abstract

**Introduction:**

Smart Phone Chat Apps (SPCA) is an integral part of people’s daily routine including orthopaedic education. SPCA facilitates efficient communication and learner-based management especially now as remote flexible learning is becoming the new norm in this COVID-19 pandemic medical training. The study described the use of a chat app (Viber) as experienced by residents and consultants in the Section of Adult Orthopaedics of the institution of the principal author. It described the mode and dynamics of the chat discussion amongst its participants, its perceived usefulness in teaching and learning specifically its relevance and applicability, its potential as a supplementary assessment tool, as well as its perceived effects.

**Materials and methods:**

This is a phenomenological study and strictly adhered to data privacy. The principal author conducted a participant observation of residents’ three-month clinical rotation at the study site. Mobile phone screenshots of the chat interactions and focus group discussions with consultants and residents were done. Residents were also requested to complete a questionnaire. All qualitative data were iteratively content analysed and emerging themes were summarised using NViVO-12. Frequencies and percentage distribution were used to analyse quantitative data.

**Results:**

Respondents included eleven senior, four junior residents, and nine consultants. Results show that SPCA is a useful, applicable, and relevant teaching and assessment tool. Influxes of multiple ideas per case were discussed real-time as the chat exchanges and interactions helped in the planning of the surgical management and eventual decision-making. SPCA also served as an effective surgical case log and online library, as well as an efficient, rapid, economical mode of information dissemination. The residents reported that it helped in developing their emotional maturity through self-reflection and self-criticism in the performance of their cases. The consultants concurred and added that they too were updated professionally in certain fields in orthopaedics. **Conclusion:** The SPCA is a helpful, relevant, and acceptable adjunct teaching and learning tool for clinical teaching and can be, to a certain extent, a supplementary formative assessment tool of the resident’s communication skills, work ethics, initiative, and diligence.

## Introduction

The use of the hand-held mobile smart phone is now a global basic and standard gadget in daily life. In the Philippines, there are 70.7 million smart phone users as of 2019 and is projected to be about 89.48 million by 2025^1^. This significant use of smart phone and its applications (apps) in the global social market has received keen interest among the proponents of e-learning such that it has been adopted and incorporated into medical education^2^, healthcare professions, and orthopaedic residency training.

The interdependent use of mobile phones for medical practice, learning and teaching as well as its enhanced use in daily living has been well discussed^3-5^ and a very close link in all these aspects has been noted. Through the wide use of smart mobile phones, medical practitioners, including the faculty of orthopaedic surgery, have been partly teaching their trainees by chat discussions. These trainees get to know more about their cases exemplifying a cycle of teaching and learning and clinical practice both as part of their continuing medical education (CME) and professional development (CPD). Subsequently, as both the trainer and trainee build up their experiences using mobile smart phones in their daily lives, they develop an inter-dependent cycle of a mobile phone-enabled teaching and learning and a mobile phone-enhanced practice in daily living.

Several studies have noted that smart phone chat applications (SPCA) show effective and efficient method of enhancing patient care and medical education. Its benefits include the convenience of a hand-held mobile gadget, which is essentially a lightweight device functioning as a computer capable of multiple tasks, with efficient communication venues through calls and emails to gather more information even prior to the actual patient-physician contact. This practice enhanced the quality of patient care through the further use of medical apps to keep track of the patients’ data^6^. The use of mobile phone amongst orthopaedic surgeons have also significantly increased especially in relation to clinical mobile apps^7-9^.

Among the SPCAs, the Viber chat app is more popular in the Philippines. At the institution of study, the app was started in June 2016. The Viber group chat was composed of residents and consultants of the Section of Adult Orthopaedics. Due to the heavy patient and surgical case load and the occasional lack of quality time for a more extensive case discussion in the Pre-operative and Post-operative Conferences, the Viber chat app became an adjunct venue to informally discuss cases for surgery, paving the way for a better look into a high proportion of the elective cases, including last-minute admissions that are to be added to the surgical list for the next day. In 2017, the entries were modified to conform with the Data Privacy Act^10^. Over the last five years, the usage has evolved in terms of data entries and quality of information exchange unique to the team. It is deemed to be timely and relevant within the current context, and more emphatic during this pandemic as it has become supplemental to the virtual telemedicine conferences and webinars. The use of SPCA in the residency training also facilitated the institution’s implementation of an outcome-based education program where technology apps facilitate not only the teaching and learning but also the sharing of clinical references and salient patient data for joint management.

The use of the Viber chat discussion for teaching and learning in the institution of study has never been systematically studied. This study aims to investigate how the smart phone chat apps is being used as a venue for teaching and learning as well as assessment of residents in orthopaedics. Specifically, the study (1) described the manner, mode, and dynamics of interactions in the SPCA used for teaching and learning, (2) described the structure and essence of the discussion amongst the consultants and residents, and how these relationships evolved over time, and (3) investigated how the residents perceived the SPCA in terms of its usefulness and acceptability as a supplementary method for assessment of clinical competence.

## Materials and Methods

This is a descriptive qualitative study following the phenomenological approach. This research design focuses on describing the structure and essence of the experiences of the participants. The complexity and intricacies seen in the dynamics of chat interactions among orthopaedic consultants and residents and the diverse perspectives shared could only be captured and adequately described through this research design.

Participants were selected purposively as those who rotated in the Section of Adult Orthopaedics of the study site based in the Philippines. These included all the nine consultants, eleven senior, and four junior residents who completed the clinical rotation from January to April 2020. Both groups were oriented face-to-face on their role as respondents of the study during one of the regular conferences. Their informed consent form was also explained and secured. Participants were assured of privacy of data that would be collected in the study. The principal author explained to all respondents that their identities would be anonymised using coded pseudonyms, e.g., Marvel superheroes for consultants and Disney characters for the residents.

For the first two research objectives, this study used a combination of descriptive qualitative data collection procedures. The principal author served as a participant observer in all the conferences and academic activities of the section, including the actual exchange of chats in Viber. Three focus group discussions (FGD) with the residents and one round of the FGD with consultants via Zoom were conducted and video recorded. The FGDs were conducted by trained moderators using a set of guide questions presented as Appendix 1 (for residents) and Appendix 2 (for consultants). The residents were randomly grouped according to the training levels (years of training) but were kept to a maximum of five participants to ensure active and maximal participation. The FGDs were held in a day and time convenient to most. Furthermore, screenshots of messages posted in the group chat by all residents and consultants within the data collection period were collected to corroborate the data observed during the conferences. For the last research objective, all residents were requested to accomplish a pre- and post-rotation questionnaire. This questionnaire was pilot tested with four residents six months before the actual study was conducted to ensure validity. Appendices 3 and 4 present the questionnaire.

All descriptive qualitative data were organised, and content analysed according to recurring themes. Iterative analysis of the FGD transcriptions were done, corroborated with the principal author’s field notes and screenshots collected. The form, manner, dynamics of interactions and overall structure of the SPCA were inferred and verified using the NVIVO-12. Quantitative data was analysed using frequency counts and summary statistics.

## Results

The profile of the respondents in the study group included fifteen residents of varying year levels and seventeen consultants. All the fifteen residents and nine consultants consented to participate in the study. Out of the nine consultants, only one is female, seven are part-time, six are tenured Medical Specialists, and three are temporary Medical Officers. Their mean age is 43.75 years. All of them are Fellows of the national orthopaedic organisation specialising in Arthroplasty, Foot and Ankle Surgery, Sports Medicine and Arthroscopic Surgery, and Tumours. The consultants are all digital natives, having initially started out using smart phones rather than analogue phones and are well versed in the use of Facebook Messenger, Viber, and other modes of internet-based technology platforms.

The residents, on the other hand, are all temporarily employed doctors undergoing specialty training. They hold a Medical Officer III position renewed yearly pending completion of promotion requirements. There are four females and eleven males, four second year and eleven are fourth year levels of training. Mean age is 30.4 years. As millennial learners, they are well-versed in all platforms of digital technology, including smart phones and chat apps.

**Fig. 1:**
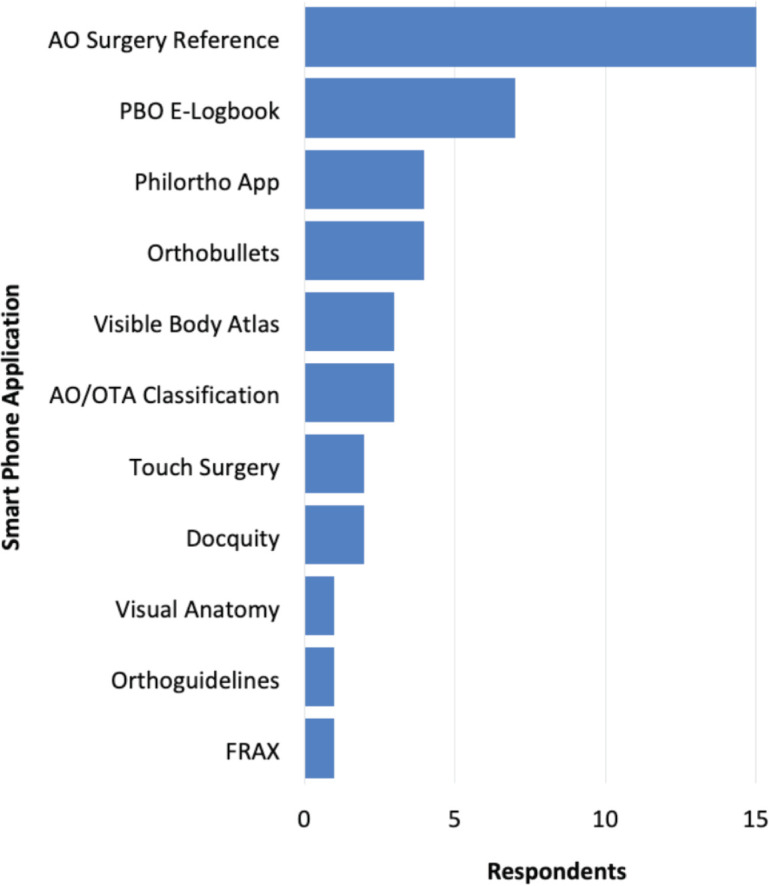
Frequency of common applications used by residents in their devices.

All the resident participants used smart phones for daily use (with an iPhone being most common followed by an android [Samsung]). Fig. 1 presents the orthopaedic apps that residents use in their smart phones, and typically, on the average, make use of about three orthopaedic apps, with the AO Surgery Reference, the e-logbook of the national examining and certifying Board, and OrthoBullets as the three most common. The e-logbook of The Board is the tool being used for program evaluation and resident credentialing.

**Fig. 2:**
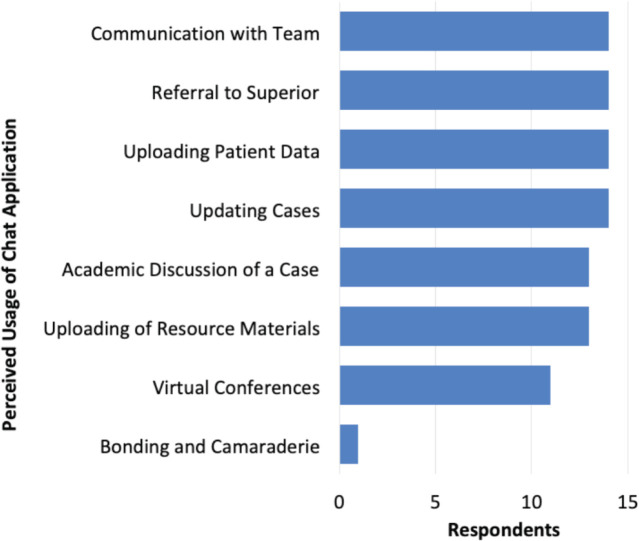
Frequency of respondents’ perceived usage of SPCA.

In terms of use for the SPCA, the resident participants reported that they have been using Viber for a lot of reasons primarily for communication with the entire Adult Orthopaedic Team, referral of cases, updating of a patient’s clinical status, and uploading of one’s case for pre-operative and post-operative discussions. These modes of usage all gathered the same frequency (n=14). The other reasons for perceived usage of the SPCA include academic discussion of the cases and uploading of relevant resources to support the conversation. Only one respondent wrote that the chat app was being used for bonding and camaraderie. Fig. 2 presents the frequency counts of each of these reasons.

The authors looked at the mode and dynamics of chat interactions among participants, development of relationships, and how the interactions are valued. Compared to their male co-residents, the female residents, regardless of year level, were noted to be more articulate, asked and followed up more intensively their cases, and were more diligent in the completion of their data entries. All the residents found the Viber group both relevant and applicable to their training and appreciated the inputs of the consultants, who have been helping and influencing them in the preoperative preparation of their cases and decision-making of the surgical plans by getting a lot of ideas and comments that contribute to the planning of the case. After the initial self-directed study, the case is posted in the Viber chat discussion thread, then Socratic questions usually follow that gravitate into an open, free-flowing discussion. This gives the residents an opportunity to look at the case more profoundly and study the problem before the weekly formal actual live interactive conference. The different ideas help the residents in further planning and deciding for the surgical management of the patient, from pre-operative to the post-operative and rehabilitation phases of treatment. In the process, the residents’ viewpoints are enhanced, and their horizons broadened. Excerpts from the respondent’s accounts show the SPCA as a venue for generating multiple ideas and inputs:

Goofy:*“this (referring to the chats) triggers a lot of questions and concerns in the discussion. We are then prodded to read and answer or defend some of the ideas (of the other consultants or co-residents [it’s way better and preferable to have a free-flowing discussion as a lot of ideas come in] from almost everyone...consultants and residents)”.*

Pluto: *“...we get to discuss or to pitch in our opinion...(and) it gives a fresh set of eyes to analyse the information as well as the different strategies; it provides a space for discussing different opinions. So, it’s a big help for complicated cases”.*

Minnie Mouse: *“... (For me), for pre-op particularly, (is more helpful). For example, sometimes you can’t decide whether to proceed with Plan A or Plan B, but upon discussion and exchange of ideas and plans, we can come up with a consensus...... So, the important thing is to recognise the advantages and disadvantages of a particular approach and I think our Viber discussion really helps me in that”.*

**Fig. 3:**
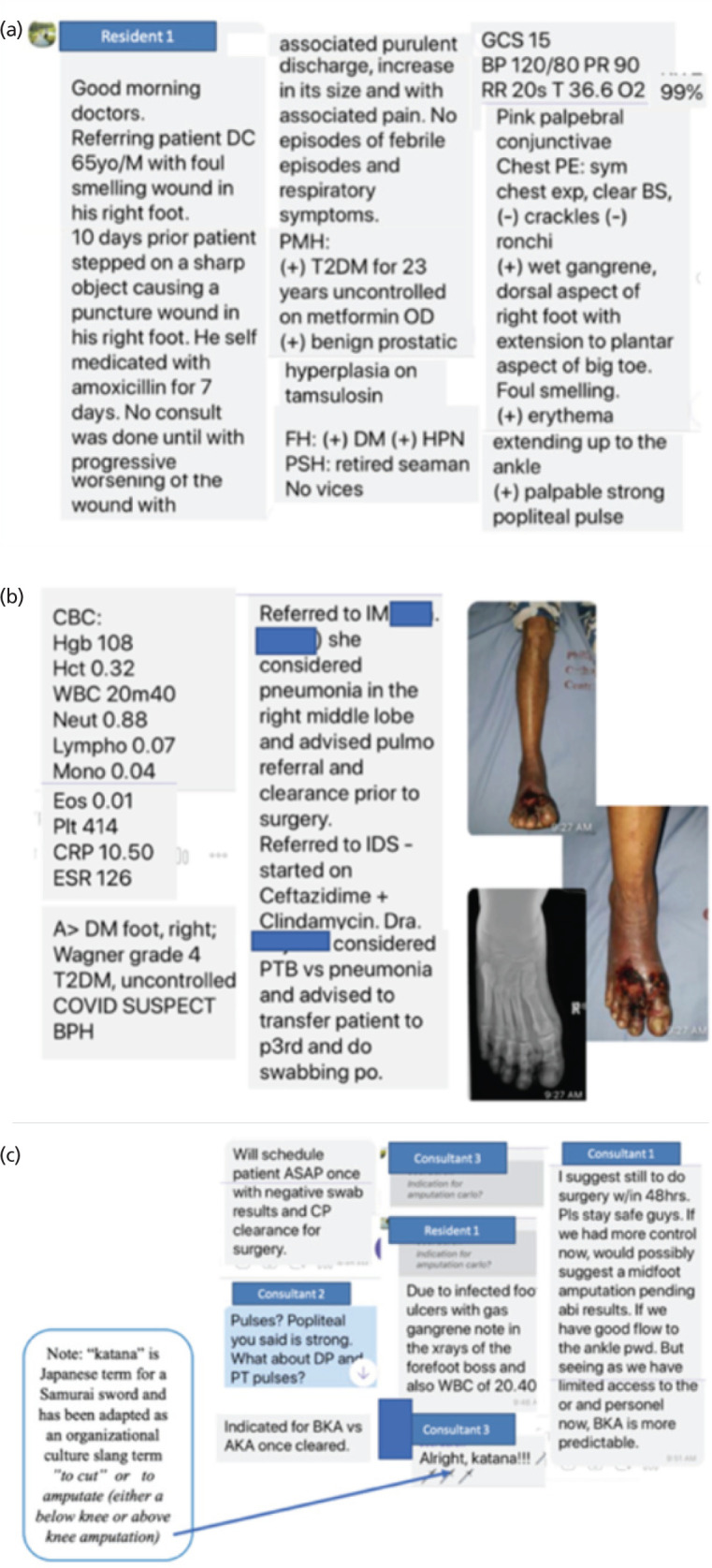
(a) Screenshot showing initial presentation of the patient’s basic information by a resident to the whole group. (b) Screenshot showing results of various consultations with the other medical specialists. (c) Screenshots showing interchange among orthopaedic consultants leading to the eventual decision for surgical intervention.

Fig. 3, illustrates chats from a resident reporting basic patient information, referrals made to consultants, that contribute to the decision-making of an urgent case needing surgical management. The thread was edited to follow the sequence of discussion.

The consultants were also asked about the value of the SPCA to augment to the Section’s live face-to-face conferences in a more straight-forward manner. They relayed these comments:

Captain America: *“I like that the pre-op and post-op discussions are also in Viber since we only have pre-op conference live face to face once a week, we don’t have the luxury of time. So here, we have a venue where we can do this every day without us being (physically) together. So, it’s*

*an extension of the round-table table discussions that we have. ... if we miss out on something in our sit-down conferences, then they can fill us up on those.... especially now since most of our conferences are online”.*

Hulk: *“I think the Viber messaging is a valuable tool in teaching our residents especially in this time of COVID-19”.*

The residents reported that the chats direct the discussion and contribute to make a surgical case log. Since part of their assessment includes a portfolio, the use of the SPCA, with all its data entries for the pre-op and post-op discussions helps them make a preliminary e-portfolio. It was also cited that the compilation of cases becomes an “online reference library” serving as possible bases for their decisions in similar cases in the future. They affirmed the value of SPCA through the following excerpts:

Pluto: *“.... if you need to go back to what the Boss (Consultant) said and his recommendations exactly...... you can go back and go thru the messages, one by one, as well as the photographs, radiographs, and whatever is recorded there. It is a good tool to collate things and, at the same time, go back on the things that were already discussed”.*

Donald Duck: *“.... for me (the biggest advantage with) Viber is it’s like an ‘online library’.... it’s a recording of meetings and discussions, in a printed form of a chat.... it keeps record of our procedures. This is very useful (as) you can go back to the previous discussions, to clarify some things. What I even do is, sometimes when I have a difficult case, I also look back at the previous discussions, previous radiographs, opinions, and comments from the consultants, and it’s very helpful”.*

**Fig. 4:**
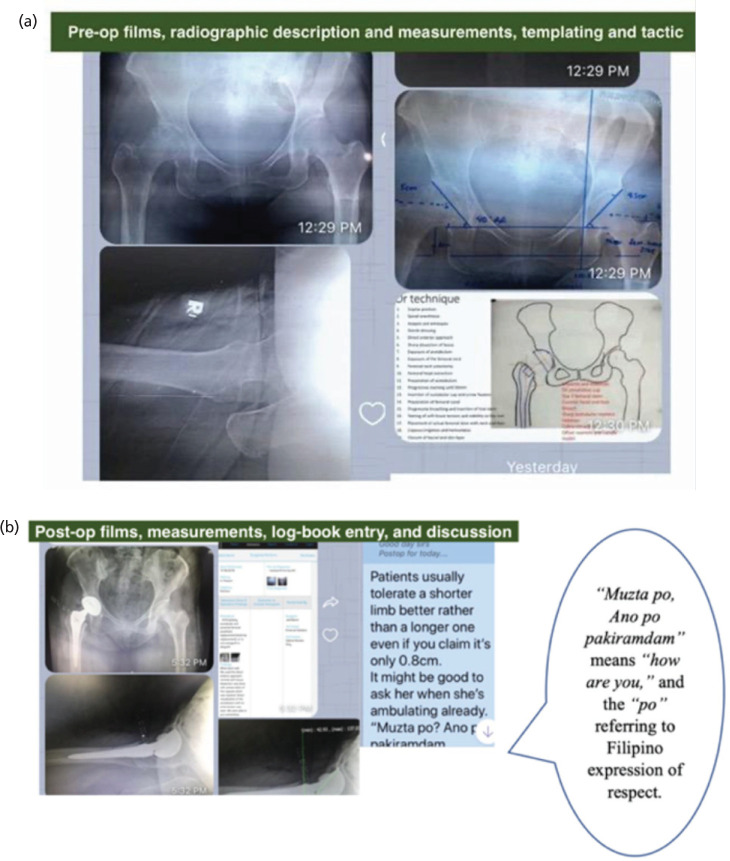
(a) Screenshots showing pre-op planning, including templating and surgical tactic. (b) Screenshots showing post-op discussions of the same case, including some comments on postop management, and entry into their eLogbook.

Fig. 4 presents screenshots of residents’ own work on surgical planning and templating (or radiologic measurements and objectively determining what orthopaedic implants to use). These are again logged in the PBO e-logbook, simulating an e-portfolio. The screenshots show an example of how a link or even an article can be uploaded for additional references and resources for more in-depth reading.

The chat discussions involve continuous exchange and feedback that enabled residents to self-reflect. During postoperative conferences, they reflect on (1) ‘What do you think went well in your surgery?’ and (2) ‘What will you do differently next time?’ These questions gave the residents opportunities for metacognition and the time to reflect more on one’s case, outline their salient learning points, and how to perform better next time. Residents reported that they realised through an open chat discussion, how to develop their own thought processing and critical thinking, and to learn from the shortcomings and deficiencies of others. They expressed appreciation and valued this part captured in the following:

Prince Charming: *“.... (that’s the beauty of the) post-op (discussion) (It doesn’t mean that your work shouldn’t be criticised anymore). So, the benefit of it is that everybody gets to criticise their own work. And that they (Faculty) get to tell you and comment if something is good, or something is wrong”.*

Sleeping Beauty: *“I think focusing on the good aspects of the surgery like ‘what went well’ is important as it directs you, as a surgeon, on your strengths, how were you able to achieve this, etc. I can also appreciate (what was eventually shared to you) “what would you do differently next time” because it helps me ask questions to think out of the box or in a certain framework, as we enter the OR next time”.*

Pocahontas: *“...There is usually an inventory of what is being done, (so one gets to see his own work which you initially thought was already good, yet it turned out to be wrong or deficient)! ... And then, (since there are several critics, you also get to learn from them). It’s being done in a very constructive way”).*

**Fig. 5:**
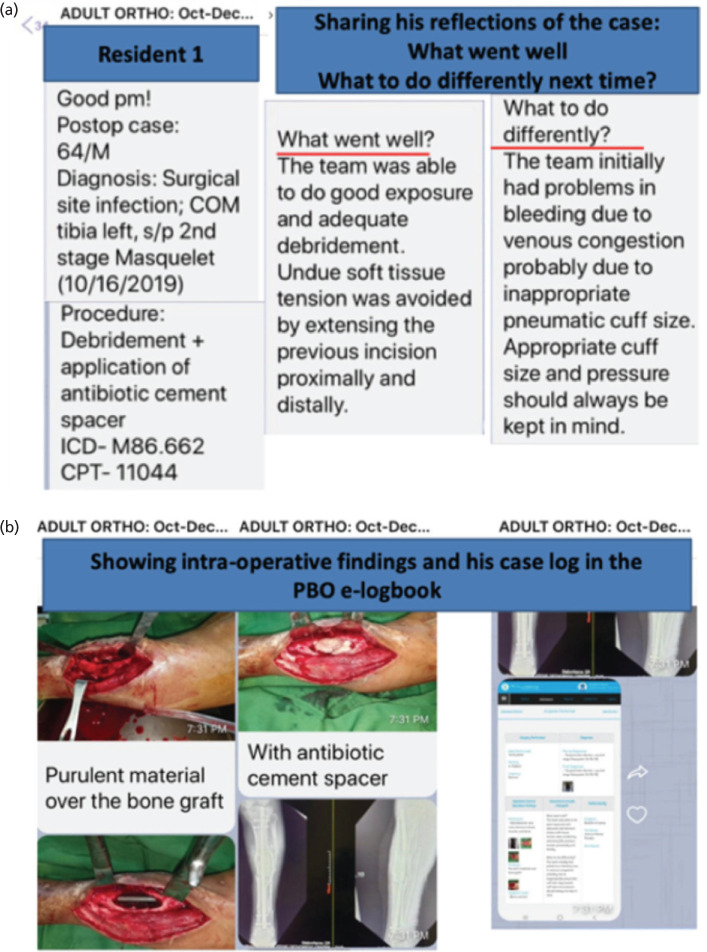
(a) Screenshots of Viber app used as a venue for personal reflections for comment by the group. (b) Screenshots showing intra-op pictures and entry into the eLogbook incorporating consultants’ feedback on the case.

Fig. 5 presents how one resident gives his reflections and how feedback is given. It also shows how his plan is supported by uploaded literature.

The continuous flow of chat apps was also appreciated by the consultants in terms of updating themselves on selected areas in orthopaedics. The following excerpt shows this theme of updates and continuing education.

Captain America: *“... for the faculty, it helps (since we are) sub-specialty-based.... like for example, being a Tumour Surgeon, I learned Foot and Ankle Surgery, Sports, from the other specialty consultants, simply because (I haven’t been updated much in those areas already). So, with these kinds of discussions, at least (one gets to be refreshed and updated in the other sub-specialty areas)”.*

**Fig. 6:**
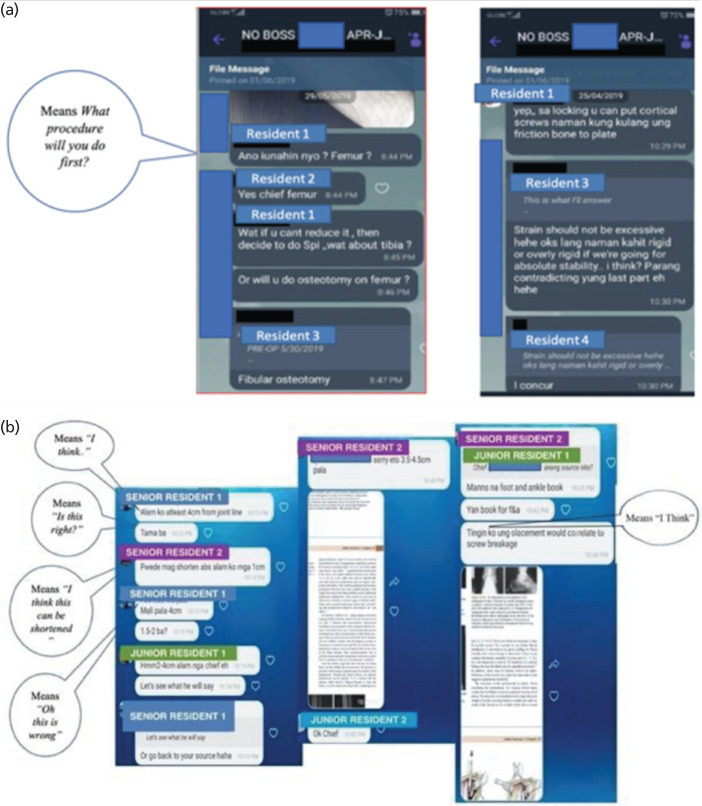
(a) Screenshots of discussions among residents of the same year level as evidence of peer teaching and collaborative learning occurring in the *No Boss* group chat. (b) A similar set of screenshots as evidence of collaborative teaching among Junior and Senior Residents, including attachments of literature support for such a decision.

The residents have also formed their own chat group within the Section of Adult Orthopaedics (AO). These are the “AO KIDS” or “AO NO BOSS” group that do not include the consultants. This is a parallel running thread exclusively amongst themselves. The residents consult one another, having a common scenario of the seniors sharing with one another their own experiences on a similar case and / or a junior freely asking his senior and even all the seniors sharing their experiences and jointly teaching the juniors, without the consultants. The screenshots in (Fig. 6) show this active exchange of collaborative learning.

The residents themselves through Mickey Mouse said *“…. Viber (is) used as a supplemental tool for learning; it’s not the main avenue for achievement of learning; still the main source of learning is through our own time reading but as an additional tool, it is really very helpful.”*

There is also an assessment that the Viber chat group is like a double-edged sword having both advantages and disadvantages, the latter of which is discussed and elaborated by Pluto.

Pluto: *“Yes .... a “double edged sword” …First, (it’s about the) assessments on the advantages and disadvantages.... for the pre-op discussion, (we send 4 cases for pre-op discussion but sometimes, what is thoroughly discussed is one case - just the difficult one) Second, there are people (who are quick to respond) so you tend to manage the patient timely; but (sometimes, there are also people who are not too quick to respond causing undue delay in patient management, especially in urgent cases). So that’s what I meant with a ‘double edged sword’”.*

The residents themselves have realised that the SPCA can be a way to assess their own critical thinking as well as their own attitude. The chats help them to reflect, think deeply, and realise their shortcomings.

Pluto: *“... if you criticise and check our own work (my own and other resident’s) and cross it with the opinion of the consultants.... it triggers some critical thinking and analysis (especially if there are questions raised) about complicated cases (such that) it really makes you think.... For example, (in the) pre-op discussion (you posted your data and think that you’ve got all the areas covered thinking there are no more questions) then suddenly (a question from a consultant pops up), ‘So how will you do it?’ (The question makes you think.... how am I going to do it? [how am I to address the situation?]’”.*

Consultant Thor: *‘I like Viber (on) how it’s being used by the team... you get to see how the residents are thinking, their trend of thought, logic, the way they process information, and how they’re able to reason out for their cases”.*

Resident Mickey Mouse explained *“It also develops the residents’ attitude; you learn how to be ethical and as a training resident, you learn how to accept criticisms. We learn how to give inputs, and essentially, some of the inputs from our seniors, peers and even our juniors, (that is constructive), you learn how to incorporate them in your work”.*

Pocahontas added: *“.... attitude and behaviour are very important aspects that are reinforced by the Viber communication.... we are forced to self-reflect and I truly believe that anyone who is developing and honing their skills, self-reflection after doing such procedures is very important, or maybe even much more important than pre-op planning. .... it’s the only way you will learn about what you did; how you can improve upon yourself in the future”.*

Thor said: *“I think Viber can help assess the residents’ attitude in the manner how they refer the patients through the text… how respectful they are, the completeness of their data, the promptness of their response, their initiative to post updates, or suggest the plan even without asking them”.*

Black Widow: *“.... for assessment of attitude, if we are talking about judging work ethics, I think we can. For example, a good resident with outstanding work ethics presents a pre-op case with proper and thorough PE, assessment, and a well thought of plan backed up by updated and related literature. .... we can check on their record keeping skills, speedy referrals, and we can counter-check on how they follow instructions”.*

Iron Man: *“I’m more into interaction.... it’s easier to “read” these residents if I see them work or how they answer questions...”*

## Discussion

The participants are mostly Generation X and Millennial learners and as digital natives, all are very comfortable with the SPCA. Viber has emerged as the dominant chat app in the country because of its inherent features and its ease of use. This has led to an easy adaptation and early comfort of the participants with the platform, including the more senior faculty who are digital migrants. As to the learning styles, an orthopaedic surgeon as a converging learner, primarily a “thinker and doer” is observed to be correct^11,12^. It is keenly observed and noted that it is common for the resident learner to frequently state, “I can do this!” after watching a procedure.

Referring to Kolb’s learning styles^13^, the residents after much “individualised thinking” (abstract conceptualisation) during the augmented SPCA remote asynchronous venue, participate freely in pre-operative conference discussions. Then the next day, they implement what they learned by “doing” the case either alone or with faculty guidance, or maybe as a surgical assist (active experimentation). Abstract conceptualisation continues through the post-op conference especially after further feedback and eventually builds on this experience. The next time they encounter a similar case, they are repeating the learning cycle, emphasising once again active experimentation.

The SPCA has been noted to be a useful teaching-learning platform of clinical teaching in residency training. All the participants found that the Viber chat messenger provided a platform to easily foster communication for patient referral, post images of both the gross pictures of a patient and their imaging studies (radiographs and even ultrasound, CT, and MRI). These facilitate academic discussions, exchange of ideas, and share links to resources and other relevant educational information. The study also shows that the SPCA is in conformity with the global standards set by the World Federation for Medical Education as to the need to be competent in information and communication technology (ICT) for better communications, systems-based practice, self-directed learning, and practice-based learning^14^.

Regarding communications and patient referrals, this study had similar findings made by De Benedictis *et al* where a resident is encouraged to seek help from his peers and superiors^15^. Residents feel more at ease referring to their consultants through Viber than a personal live face-to-face encounter, helping minimise the hierarchy difference in medicine. The study shows that the SPCA has impacted practice in several aspects. It is a true and real-life example of how the mobile smart phone has revolutionised and enhanced the participants’ lifestyle with the consultants’ daily life in terms of work readily facilitated by patient referrals, monitoring and the resident’s experiential learning in orthopaedic education. The continuous exchange of chats led the residents to become more and more reflective, forcing them to process, critically think, and decide, thereby honing them to be self-directed responsible learners. The SPCA has now become an integral part of one’s mobile phone-enabled clinical practice, mobile-enabled-teaching and learning, and mobile-enabled daily life; truly mobile smart phones is a device that a healthcare worker must learn to adapt to as it is here to stay^3-5^.

In today’s pandemic, the mobile smart phone has become a necessary tool for access to resources that may not be within easy grasp, of which teleconferencing, and webinars have now provided, and with the ease of communications through this gadget, it has further enhanced the learning experience. The study provides evidence that the participants have shared a very positive outlook on the use of the app for their daily work and activities. Overall, the research has shown similar findings made by previous authors^2,6,11,15-22^.

The study also presents the SPCA as an illustrative example of the Technological Pedagogical and Content Knowledge (TPACK) at work^23,24^. The integration of content in orthopaedic training is reflected in the exchanges of chat apps. The essence of the training delivered using the SPCA Viber platform reflects the integration of content and technology, while the level and dynamism of the exchanges reveals the pedagogic component in the form of “online conference” as the new version of the typical case and endorsement conferences.

The TPACK components as seen in the study also reflect the actual direction, design, and delivery components of outcome-based education (OBE) in orthopaedic residency training. The learning outcomes refer to direction and are not just seen in terms of the content of the chat apps but more importantly on the way these chats are communicated with all the participants. Both the design and delivery components of OBE reflect the integration of ICT, the wide scope of outcomes expected from the residents developing higher order thinking skills of analysis, evaluation, and synthesis seen in residents’ clinical decision making. As to the use of the SPCA as a supplement assessment tool, it has revealed mixed perceptions. It could be helpful in gauging a resident’s attitude in diligence, ethical work, and communication skills. The consultants, however, mostly believe that it can serve as a formative assessment supplement or an adjunct but cannot be a substitute for a face-to-face assessment.

The SPCA also has disadvantages. Chats continue to be online 24 hours a day, creating a perceived increase in workload, and disruptions in relationships^15^. Issues on time management and its unintended invasion of personal privacy can be seen in the screenshots made at night, even on weekends. Cognisant of these, the residents thought of separating the pre-op and post-op postings and discussions and allocated a time frame for such. Most agree that 9:3010:00 PM should be a “lights off” for the all the Viber posts unless it is an urgent or emergent patient referral.

In OBE, documentation refers to the collection of workplace-based and authentic proofs of performance^25,26^. In this study, the exchanges of chat apps between and among participants, from data collection on a particular patient’s case (reflective of residents’ competencies in history taking and gathering of pertinent data), all the way to choosing the appropriate management plans and actual execution of the correct surgical procedures, revealing their decision-making competencies are real-time and authentic proofs of their progress in the training. How they receive, reflect, and act after the series of chats are more than adequate documentations of their performance to help the consultants in assessing their progress in the training.

## Conclusion

The Smartphone Chat App (SPCA) specifically Viber, in orthopaedic residency training is an adjunct teaching and assessment tool. Overall, the Viber chat discussion has further enhanced connectivity amongst the participants and facilitated communication within the Section. The added benefits of the instant messaging platform such as a record of discussion and the ability to share data, such as gross medical pictures and radiological imaging, have been very useful and made possible through a hand-held computer, the smart mobile phone. The use of chat apps (Viber) as a tool to discuss patients’ problems and mode of management in the clinical environment of a local orthopaedic setting in a big training institution has proved to be a good adjunct tool for teaching and learning and, to a certain extent, even as a supplementary tool for formative assessment of the trainees. The SPCA is appreciated as a tool documenting a resident’s communication skills, work ethics, initiative, and diligence. Overall, the SPCA has been an exemplary tool in expediting patient care.

For recommendations, this study was started a few months before the pandemic and completed in late 2020 while the Philippines was still amid COVID-19. Medical services at the study site did not stop and the residents handled the Section with limited face-to-face and mostly online supervision from consultants. The study is cognisant that aside from smart phone chat apps, other ICTs were used by the institution to ensure quality health service delivery and residency training. This study recommends that residency training programs from other fields of specialisation are explored, not only in terms of investigating the use of chat apps but also other complimenting technological applications, how these are applied to reinforce the human elements like consultant supervision and direct patient interactions, both for the acquisition of knowledge, skills, and attitudes. Now that it is also becoming apparent that online learning will continue even after the pandemic, studies that will search for the right blending of technology, integration of content, and pedagogy to facilitate learning of clinical skills in residency training will be most timely and justifiable.

## Appendices

### **Appendix 1:** Guide Questions for the FGD for Residents.

1. What do you think went well with the use of the Viber chat group?
  - Did you find it helpful for you? In what way?
  - Do you think it helped you in the preparation of your cases? Preop? PostOp? How? Why? Why not?
  - What are your other learnings through this chat group discussion? Can you be more specific? Would you like to elucidate on it or further elaborate that? Why? How helpful was this for your rotation?
  - Aside from knowledge, do you think this chat discussion group helped you in the other domains of learning? Like? Why? Why not? How? Can you please elaborate?
  - Do you think this Viber chat discussion group is something you’d like to be used in your other specialty service rotations? Why? Why not?
2. What should we do differently regarding the Viber chat group next time?
  - Why? How?
  - Should we add / delete some of the data that we enter? Which? Why? Why not?
  - Should we have a deadline as to when we should do our Viber postings? Why?

Thank you for your warm participation. We truly value your inputs and really look forward to inviting you soon to another focused group discussion should there be a need to elaborate on some of your comments.

### **Appendix 2:** Guide questions for FGD for Consultants.

1. Do you think this Viber chat group can be an adjunct tool for teaching the residents? Yes/No? Why/Why not? How?
2. Do you think this Viber chat group can be an adjunct tool for Assessment of the residents? Yes/No? Why/Why not? How?
3. Should we add / delete some of the data that we enter? Which? Why?
4. Do you think this is something we should continue?
  If yes, do we have to modify or even stop some ways on how we are doing it? If no, why?

Thank you for your warm participation. We truly value your inputs and really look forward to inviting you soon to another focused group discussion to further elaborate on some of your comments.

### **Appendix 3:** Perceptions of the Smart Phone Chat Apps Pre-Rotation Questionnaire Instrument

Greetings!

We will be conducting a study on the use of the Viber Chat Group as a training and an assessment tool for the Orthopaedic Residency training, specifically the Adult Orthopaedics Service. Your honest response will be highly appreciated.

Name: (optional) ______________________________________________

Year Level: (please encircle) 1 2 3 4

1. What smart phone do you use currently? Please specify the model
  - [ ] Samsung
  - [ ] iPhone
  - [ ] Huawei
  - [ ] Oppo
  - [ ] __________________________
2. What specific orthopaedic apps do you have in your smartphone?
  __________________________________________________________________________________________________
  __________________________________________________________________________________________________
3. Do you have prior experience in the use of a chat group for your personal orthopaedic education?
  - [ ] Yes, I have. Please proceed to No. 7
  - [ ] No, I don’t have. Please proceed to No. 8
4. How was this being used? For what? (You may check more than one)
  - [ ] communication amongst the members of the team
  - [ ] referral to your superior (Chief Resident or Consultant-on-Call)
  - [ ] uploading of images of your patient
  - [ ] updating of a case, especially problematic or complex case
  - [ ] chat group academic discussion of a case
  - [ ] virtual conferences (e.g. Pre-op conference)
  - [ ] uploading of resource materials (e.g. PDF articles)
  - [ ] others, please specify: __________________________
5. If you had a prior experience in using the Viber chat group, how did you find it?
  - [ ] Very helpful in my training
  - [ ] Somewhat helpful in my training
  - [ ] Not at all helpful in my training
    Please elaborate.
    __________________________________________________________________________________________________
    __________________________________________________________________________________________________
6. If you haven’t had any experience in using the Viber group, do you think it will help you in terms of usefulness in comprehending the case you have at hand?
  - [ ] Yes
  - [ ] No
7. What are your expectations from Question No.6?
  - [ ] Relevance
    - Very relevant to my education
    - May be somewhat relevant to my education
    - Not relevant for me at all
  - [ ] Applicability
    - I will find this very useful, and I will continue using it freely in my education
    - I will probably find it somewhat useful and may apply it occasionally
    - I will probably find this Viber chat group not useful at all for me
8. What do you think are advantages of this chat group app in the discussion of cases especially the Pre-op and Post-op conferences?
  __________________________________________________________________________________________________
  __________________________________________________________________________________________________
9. What do you think are its disadvantages?
  __________________________________________________________________________________________________
  __________________________________________________________________________________________________
10. Any additional comments or viewpoints?
  __________________________________________________________________________________________________
  __________________________________________________________________________________________________

Thank you for your warm participation. We truly value your inputs and really look forward to inviting you soon to a focused group discussion to talk about this Viber app further.

### **Appendix 4:** Perceptions of the Smart Phone Chat Apps Post-Rotation Questionnaire Instrument

Greetings!

We have just concluded a study on the use of the Viber Chat Group as a training and an assessment tool for the Adult Orthopedic Residency training. Your honest response will be highly appreciated.

Name: (optional) _____________________________________________

Year Level (please encircle) 1 2 3 4

1. How did you find the use of the Viber discussion group in terms of the following:
  - [ ] Relevance
    - I found it to be very relevant to my education
    - I found it somewhat relevant to my education
    - Not relevant for me at all
  - [ ] Applicability
    - I found will find this very useful and I will continue using it freely in my education
    - I found it somewhat useful and may apply it occasionally
    - I found this Viber chat group to be waste of time and not useful at all for me
2. Based on your experience, what are the advantages of these chat group apps in the discussion of cases?
3. Based on your experience, what are its disadvantages?
  __________________________________________________________________________________________________
  __________________________________________________________________________________________________

Thank you for your warm participation. We truly value your inputs and really look forward to inviting you soon to a focused group discussion to talk about this Viber apps further.
